# Selective phonotaxis of female crickets under natural outdoor conditions

**DOI:** 10.1007/s00359-014-0881-7

**Published:** 2014-02-01

**Authors:** Stefan Hirtenlehner, Heiner Römer

**Affiliations:** Department of Zoology, Karl-Franzens-University, Universitätsplatz 2, 8010 Graz, Austria

**Keywords:** Phonotaxis, Female choice, Acoustic communication, Field cricket, Decision making

## Abstract

Acoustic mate choice in insects has been extensively studied under laboratory conditions, using different behavioural paradigms. Ideally, however, mate choice designs should reflect natural conditions, including the physical properties of the transmission channel for the signal. Since little is known about the discrimination ability of females between male song variants under natural conditions, we performed phonotaxis experiments with female field crickets (*Gryllus bimaculatus*) outdoors, using two-choice decisions based on differences in carrier frequency, sound pressure level, and chirp rate. For all three song parameters, minimum differences necessary for a significant preference between two song models were considerably larger outdoors compared to laboratory conditions. A minimum amplitude difference of 5 dB was required for a significant choice in the field, compared to only 1–2 dB reported for lab-based experiments. Due to the tuned receiver system, differences in carrier frequency equal differences in perceived loudness, and the results on choice for differences in carrier frequency corroborate those in amplitude. Similarly, chirp rate differences of 50 chirps/min were required outdoors compared to only 20 chirps/min in the lab. For predictions about patterns of sexual selection, future studies need to consider the different outcomes of mate choice decisions in lab and field trials.

## Introduction

Numerous studies with taxa from invertebrates to mammals have documented that males with certain attributes gain a mating advantage through female mate choice (Andersson 1994). Acoustic mate choice has been extensively studied in the past, since it offers several advantages compared to other sensory modalities. The signals being involved can easily be recorded in the field and laboratory, and the acoustic behaviour is reliably elicited in response to playbacks, in which all signal parameters are under precise control of the experimenter using audio software processing. In this way, the selectivity of receivers for certain acoustic parameters has been determined for different species, providing a framework for studying mechanisms underlying species recognition and for generating predictions about patterns of sexual selection and speciation (Gerhardt and Huber 2002; Bradbury and Vehrencamp 2011).

In particular, insects are excellent model systems to study decisions based on acoustic signals (review Gerhardt and Huber 2002; Hedwig 2006). In field crickets, for example, positive phonotaxis of the female towards the calling song of the male is the first step in mate choice. During courtship and (potentially following) copulation females may further assess other cues, such as cuticular hydrocarbons, which can provide information important for species and kin recognition (Alexander 1962; Singer 1998; Wagner and Reiser 2000; Thomas and Simmons 2009; Simmons et al. 2013).

The calling song is produced by male crickets with a species-specific temporal pattern (Otte 1992; Pollack 1998). Besides field studies in which the attraction of females towards males with certain call parameters or body traits has been quantified, or even their mating success recorded (Ulagaraj and Walker 1975; Walker and Forrest 1989; Farris et al. 1998; Rodríguez-Muñoz et al. 2010), the vast majority of studies was laboratory based, and used either trackballs, walking compensators or arenas to quantify the behaviour of receivers (Gerhardt and Huber 2002 for review). Each of these approaches has its special advantages and limitations due to the freedom of movement of the females, the preciseness of control over stimulus parameters, or whether females perform phonotaxis under open- or closed-loop conditions.

However, the ability of receivers to detect and discriminate the differences in male traits which are used for their mating decisions are also influenced by environmental conditions. Although the acoustic communication channel bears some advantages over the visual or olfactory channel, acoustic signals may be strongly degraded and attenuated by physical properties of the environment. A potentially informative character, for example, the interval between sound pulses, is degraded by reflection or echoes (Wiley and Richards 1978; Römer and Lewald 1992; Römer 1998, 2001). Furthermore, background noise can result in masking of the signal or signal components (Brumm and Slabbekoorn 2005; Römer 2013). These conditions suggest that a signal that functions efficiently in the laboratory, and can be easily discriminated from another signal variant, may not be ideal in the natural environment where individuals communicate (Endler 1992). This may explain why in several frog species the preferences of females for differences in carrier frequency or intensity as observed in the laboratory are not reflected in outdoor settings (Gerhardt 1992; Dyson et al. 1992).

Although female mate choice in insects has been studied extensively in the laboratory, phonotaxis has only rarely been observed and quantified in the natural habitat (but see Mhatre and Balakrishnan 2008 for an exception with a cricket species; and Hirtenlehner et al. 2014 for a no-choice situation). Indeed, in a field study on a wild population of *Teleogryllus commodus* Bentsen et al. (2006) showed a strong stabilizing sexual selection on call structure verifying results previously found under laboratory conditions (Brooks et al. 2005). Outdoor conditions are particularly challenging for female cricket receivers, and the experimenter recording and quantifying the behaviour, for several reasons. One is the problem of sound transmission parallel to the ground which can create complicated patterns of interactions between the direct wave and the one reflected from the ground (reviewed in Embleton 1996; see also Römer 2001). Indeed, in a recent outdoor study using neurophysiological methods it has been shown that both directional and distance information can be strongly degraded for a receiver at some distance from the source (Kostarakos and Römer 2010). Furthermore, in the grassland the female is not completely free to orient straight towards a sound source due to obstacles in the transmission channel, so that forced turns might bias the walking direction more than turns induced by the auditory information. Our hypothesis is that the strongly degraded sensory information will further reduce the reliability of choices when compared with the arena or other laboratory approaches, even when the differences in signal traits are substantial. Thus, decisions based on small differences between signal parameters may be less likely under realistic natural conditions, or, alternatively, the time necessary for decision making (arriving at a certain source) could be increased relative to the same task in the indoor arena.

The aim of the present study, therefore, was to perform quantitative phonotaxis experiments on female field crickets outdoors, using two-choice decisions based on differences in the male calling song, similar to those performed under laboratory conditions. We varied values of signal traits within the range of variation of the naturally occurring parameter space of populations in carrier frequency, sound pressure level (SPL), and chirp rate. Following the argument made by Bee et al. (2012) in their study on sound pressure level discrimination in tree frogs, we consider that such tests will estimate the “just meaningful difference” (JMD; Nelson and Marler 1990), and not “just noticeable differences” (JNDs) because in behavioural experiments it is possible that the nervous system of receivers may discriminate smaller differences in signal traits under ideal conditions which, however, cannot be used for behavioural discrimination because of noise on the transmission channel. Our results demonstrate that for all three song parameters the JMD between two song models for a significant preference of females was considerably larger in the field compared to previously reported differences in classical laboratory approaches.

## Materials and methods

### Study species and phonotaxis in the field

The study was conducted between August and October 2011 and May and September 2012. We used female crickets (*Gryllus bimaculatus* de Geer) from a population kept at a constant photo cycle of 12 h:12 h L:D and an ambient temperature of 25–30 °C at the Department of Zoology, University of Graz. Crickets were fed on water gel, fish food, oat flakes and lettuce ad libitum. Last instar females were separated from males to maintain their phonotactic responsiveness. They were kept until the final moult in groups with other females, and isolated acoustically from singing males in a separate room.

We tested the phonotactic behaviour of 97 females 1 week after their final moult in a natural grassland habitat in the vicinity of Graz, Austria. The test ground was totally isolated from traffic noise due to surrounding forest and meadows (47°6′1.2348″ N; 15°27′9.7272″E). Furthermore, the only other native cricket species in this area, *Gryllus campestris*, did not sing during the time we performed our experiments, so that masking noise in the frequency band around 5 kHz was largely absent, except for occasional bird song. The height of vegetation on the testing ground was kept between 5 cm and 10 cm, which was high enough to strongly restrict females in their freedom to walk straight ahead, but nevertheless allowed reliable video tracking from above (see below). To record female phonotaxis, we mounted a custom-made sliding holder onto a steel rope at a height of 2.5 m above the experimental area, to which a SONY handycam HDR-XR155E (Minato, Tokyo, Japan) was attached. The construction allowed moving the video camera over the whole length of the experimental area via cable control, recording the female moving from the release point to the target speaker(s). The display of the camera covered the whole width of the experimental area (given by detours of the females from a straight path to the target), and the magnification was high enough to monitor the position of females within the grass for offline quantitative analysis of their phonotactic approaches, but insufficient to resolve the longitudinal body axis of the insect.

### Acoustic stimulation

For playback experiments, we digitally generated models of male calling songs with Cool Edit Pro software (Version 2.0; Syntrillium, Phoenix, AZ, USA) at a sampling rate of 48 k-samples/s. The temporal structure of a conspecific chirp was maintained in all song models, with four sound pulses of 23 ms duration separated by a constant inter pulse interval of 16 ms, resulting in a chirp duration of 140 ms. We modelled variation in male calling songs of natural populations, by varying either the carrier frequency or the chirp rate of song models. Chirps were repeated every 600 ms, 462 ms or 400 ms to produce chirp rates of 100, 130 or 150 chirps/min, respectively. In addition, five different carrier frequencies of model songs (4.0, 4.4, 4.7, 4.9 and 5.3 kHz) were used. 4.9 kHz matched the average best frequency of female receivers, and all other carrier frequencies cover the range of variation of male calling songs in natural populations of *G. bimaculatus* (Ferreira and Ferguson 2002; Kostarakos et al. 2008).

**Fig. 1:**
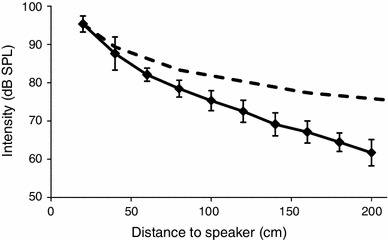
Attenuation over distance at the testing area for a signal with a carrier frequency of 4.9 kHz calibrated to 85 dB re 20 μPa at a distance of 50 cm from the speaker (mean ± standard deviation). *Dashed line* represents spreading loss of 6 dB per doubling of distance

Each song model was broadcast as an uncompressed WAV-file via an X4-Tech Bogieman IV MP3 player (Braunschweig, Germany) connected to a Kemo Germany M033 amplifier (Langen, Germany) and a full range speaker (Monacor Electronic, SP-626/8, Zwischenwasser, Austria; frequency response 600–18,000 Hz). We positioned the speaker 1 cm from the ground of the experimental area. The SPL of all signals was calibrated using a Rion NL-21 sound level meter and UC-52½ inch free-field microphone (Tokyo, Japan). Depending on the choice situation of the experiment (see below), signal intensity was adjusted to 85, 82 or 80 dB re 20 μPa, respectively, at a distance of 50 cm to the speaker at a height of 1 cm from the ground, which is about the height of the females approaching the sound source. To characterize the sound field for an approaching female, we also measured the SPL of the signal at distances ranging from 20 to 200 cm from the speaker in steps of 20 cm. The average SPL of calling songs calibrated to 85 dB at 50 cm off the speaker was about 62 dB SPL at a distance of 200 cm (Fig. 1). This is about 20 dB above the absolute hearing threshold of *G. bimaculatus* (Horseman and Huber 1994; Kostarakos et al. 2008).

### Experimental procedure

In preliminary no-choice tests on the experimental ground, we observed phonotactic approaches of females towards the speaker broadcasting a standard model song of 4.9 kHz and 85 dB SPL, when released at a distance of up to 5 m. However, to quantify and compare with similar approaches in a laboratory arena, we defined a release point at a shorter distance of 200 cm from the speakers. In two-choice tests, speakers were separated by 180 cm from each other, so the two sound sources and the release point confined an angle of about 55°.

Female crickets were transported to the testing site separated individually in plastic boxes, where they were left close to the release point for 30 min to adapt to the new conditions. Ten minutes before testing, females were exposed to low SPLs of calling songs, which they could perceive but not localize due to the closed plastic walls of the boxes. For the start of experiments, females were released by carefully overturning the uncovered plastic boxes. We defined the start of the phonotactic approach as the very moment when the female stepped out of her cage and was free to move. The end of the approach was considered when the female reached a hemicycle with a radius of 10 cm around the centre of a speaker, although 90 % of females directly encountered the membrane of the speaker. The experimenter was positioned 5 m away from the test area and controlled the match of the camera’s display detail and the cricket’s position via a SONY video walkman GV-D900E PAL (Tokyo, Japan) connected to the video camera sliding device above the walking female.

Prior to the start of a new run, we measured the air temperature near the position of the cricket. After a female finished a trial she was given a pause of at least 20 min, before we tested her again in another choice situation. When a female participated in all choice situations or when she did not show phonotactic behaviour on three consecutive days, she was returned to the lab population. Approximately 5 % of all tested crickets did not show phonotaxis at all.

Females were confronted with a two-choice situation with a standard song model (carrier frequency 4.9 kHz, chirp rate 150 chirps/min, 85 dB SPL) and a simultaneously broadcast alternative model song, varying either in carrier frequency, chirp rate, or SPL. All choices are summarized in Table 1. The sequence of choices was randomized. We also performed two-choice tests where a potential preference for higher SPL was traded against a preference for higher chirp rate. Thus, females had a choice between a simultaneous broadcast of a model song with high amplitude of 85 dB SPL, but a low chirp rate of 100 chirps/min and a model song of 80 dB SPL, but with a chirp rate of 150 chirps/min. In two-choice experiments, the temporal pattern of the alternative stimulus was presented via the opposite speaker in a time-shifted fashion, so that females were exposed to alternating chirps on the left and right speaker, respectively, broadcasting one chirp at one speaker during the inter-chirp interval of the alternative stimulus.

**Table 1:** Summarised results of all two-choice experiments

Parameters of song model 1			Parameters of song model 2			*P* value for two-choice situation (GLMM binomial model)	Number of approaches to song model 1 (left) and song model 2 (right)
SPL (dB re 20μPa)	Chirp rate (chirps/min)	CF (kHz)	SPL (dB re 20 μPa)	Chirp rate (chirps/min)	CF (kHz)		
85	150	4.9	**82**	150	4.9	0.287	32 (57.1 %) vs. 24 (42.9 %)
85	150	4.9	**80**	150	4.9	<0.001	45 (81.8 %) vs. 10 (18.2 %)
85	150	4.9	85	**130**	4.9	0.549	54 (54 %) vs. 46 (46 %)
85	150	4.9	85	**100**	4.9	<0.01	39 (70.9 %) vs. 16 (29.1 %)
85	150	4.9	85	150	**5.3**	0.0613	39 (61.9 %) vs. 24 (38.1 %)
85	150	4.9	85	150	**4.7**	0.138	9 (50 %) vs. 9 (50 %)
85	150	4.9	85	150	**4.4**	<0.001	45 (72.6 %) vs. 17 (27.4 %)
85	150	4.9	85	150	**4.0**	<0.001	30 (83.3 %) vs. 6 (16.6 %)
85	100	4.9	**80**	**150**	4.9	0.32	21 (58.3 %) vs. 15 (41.7 %)

Values in bold indicate parameters which differ between song models

To avoid a potential side bias in the outdoor setup or asymmetries in the sound propagation due to physical conditions of the grassland transmission channel, we switched the two alternative stimuli between the speakers and retested the females for the same choice condition. Importantly, we also randomized the position of the starting point on the test field within a range of 6 m day by day. Thus, females did not experience exactly the same transmission channel, such as plaques of grass bundles, in a new trial.

### Data analysis

Video files of phonotactic tracks were converted from .mts type to .avi using Any Video Converter (version 3.3.3), and two frames per second of the recordings were cut out for later analysis using VirtualDub (version 1.9.11). These frames were imported in ImageJ 1.44p (Rasband 1997–2011), which allowed a precise frame by frame identification of* x*- and* y*-positions of a female via MTrackJ plugin (Meijering et al. 2012), so that complete phonotactic approaches could be reproduced in Microsoft Excel. For the analysis of decisions of females, we carried out mixed-model analysis with language R (R Core Team 2013) and the lme4 package (Bates et al. 2012), which takes into account the non-independent nature of repeated measurements of same individuals. Since some females lost their motivation for phonotaxis after several trials, they were tested only once in a particular choice situation, whereas the majority of females were tested twice when stimuli were switched between speakers. Thus, it was necessary to fit a generalized linear mixed model (GLMM) including the animal identity as variable. For a comparison of the absolute detour length of phonotactic tracks and females’ walking speed obtained in different choice situations, we used for each individual the mean value of observations from each stimulus combination. We computed Kruskal–Wallis ANOVA on ranks, if a Shapiro–Wilk test for normality failed. To isolate the groups which differ from the others, post hoc all pairwise multiple comparison procedure after Dunn’s Method was performed. This analysis was carried out using SigmaPlot statistical software (Version 12.0; Systat Software Inc., Chicago, IL, USA).

## Results

**Fig. 2:**
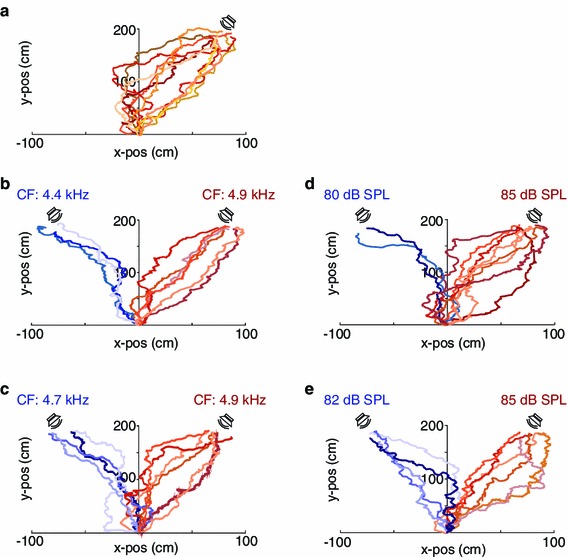
Cricket phonotaxis in the field under different stimulus situations. For each situation (no-choice (**a**); high (**b**) and low (**c**) difference in carrier frequency; high (**d**) and low (**e**) difference in song amplitude) ten examples of phonotactic tracks are provided. Notice that the quality of phonotactic tracks does not differ between two-choice and no-choice situations; for details see text. Phonotactic tracks in *red* indicate final decisions for the standard signal and tracks in *blue* indicate final decisions for the alternative song model

Figure 2 shows examples of ten phonotactic tracks for the no-choice situation and for four two-choice situations, respectively.

### No-choice trials

**Fig. 3:**
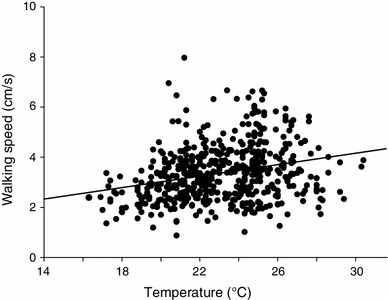
Correlation between walking speed of female *G. bimaculatus* in their natural habitat and ambient temperature at the starting point

In 44 no-choice trials, all female crickets approached the target speaker broadcasting the standard song model. For the shortest possible straight path of 200 cm, females walked an average distance of 306 cm with a high variation in the detour ranging from 34 to 264 cm (Fig. 2a). The average time taken for covering this distance was 107.3 s, resulting in an average walking speed of 3.39 cm/s. Temperature during these approaches covered a range from 18 to 26.6 °C, and with higher temperatures females approached the target faster (Fig. 3).

### Two-choice trials with differences in SPL

In a choice between two song alternatives solely differing in SPL, female crickets did not significantly approach the louder sound source, when the difference in SPL was only 3 dB (GLMM: *N* = 56, *P* = 0.287). Females approached the louder (85 dB SPL) or softer (82 dB SPL) standard model song in 32 (57.1 %) and 24 (42.9 %) trials, respectively (Fig. 2e). An increase in the amplitude difference to 5 dB (85 vs. 80 dB SPL) between the two alternatives resulted in a significant preference for the louder calling song (GLMM: *N* = 55, *P* < 0.001), with 45 (81.8 %) and 10 (18.2 %) females approaching the louder or softer sound source, respectively (Fig. 2d).

### Two-choice trials with differences in chirp rate

Similar results were obtained when testing female preference for chirp rate differences. In a choice between model songs differing in chirp rate by only 20 chirps/min (150 vs. 130 chirps/min) there was no significant preference for the higher chirp rate (GLMM: *N* = 100, *P* = 0.549), and females approached the higher or lower rate almost equally often (54 and 46 trials, respectively). When the rate difference was increased to 50 chirps/min (150 vs. 100 chirps/min), the higher chirp rate was preferred in 39 out of 55 trials (70.9 %) (GLMM: *N* = 55, *P* < 0.01).

We also traded the two observed preferences for a difference in SPL of 5 dB with a preference for a difference in chirp rate by 50 chirps/min. We simultaneously presented a model song of high amplitude and low chirp rate (85 dB SPL, 100 chirps/min) and one of low amplitude and high chirp rate (80 dB SPL, 150 chirps/min) from either of both speakers. There was no significant preference for either calling song model; females approached the high amplitude model in 21 (58.3 %) trials and the high chirp rate model in 15 (41.7 %) trials (GLMM: *N* = 36, *P* = 0.32).

### Two-choice trials with differences in carrier frequency

Four different carrier frequencies representing the range of variation in natural populations were tested against a standard carrier frequency of 4.9 kHz, which represents the mean best frequency of hearing in *G. bimaculatus* (Kostarakos et al. 2009). In such two-choice tests, females did not discriminate between the standard carrier frequency of 4.9 kHz and model songs of 4.7 kHz (Fig. 2c; GLMM: *N* = 18, *P* = 0.138). Even a difference in carrier frequency of 400 Hz (4.9 vs. 5.3 kHz) was not sufficient for a significant preference (GLMM: *N* = 63, *P* = 0.0613). However, a significant preference was observed in a choice between the standard model at 4.9 kHz and alternative model at either 4.4 kHz or 4.0 kHz. In 45 (72.6 %) out of 62 trials, females did chose 4.9 kHz over the alternative at 4.4 kHz (Fig. 2b; GLMM: *N* = 62, *P* < 0.001), in 30 (83.3 %) out of 36 trials they preferred the standard over a carrier frequency of 4.0 kHz (GLMM: *N* = 36, *P* < 0.001).

### Comparison of no-choice trials with two-choice trials

We calculated the lengths of phonotactic detours for each choice situation and compared them with the detour values obtained in no-choice experiments. There was a statistical difference between the groups (Shapiro–Wilk test: *P* < 0.05; Kruskal–Wallis test: *H*9 = 24.144, *P* = 0.004), but a post hoc multiple comparison procedure after Dunn’s method revealed that the length of detours in no-choice trials did not differ from detour values obtained in any two-choice situation. Instead, there was a difference in the groups with the highest median (trading experiment; median 121.6 cm) and lowest median (frequency test with an alternative stimulus carrier frequency of 4.4 kHz; median 76.8 cm) for detour length.

The analysis of walking speed revealed similar results. The walking speed of females in a no-choice situation did not differ from their speed in two-choice experiments, but again there was a difference in the groups with the lowest median (trading experiment; median 2.8 cm/s) and highest median (frequency test with an alternative stimulus carrier frequency of 4.4 kHz; median 3.7 cm/s) for walking speed (Shapiro–Wilk test: *P* < 0.05; Kruskal–Wallis test: *H*9 = 19.399, *P* = 0.022; post hoc all pairwise multiple comparison procedure after Dunn’s method).

To quantify the dependence of walking speed on temperature we performed a correlation analysis including every single data point, ignoring multiple testing of an individual. We found a linear correlation between walking speed (Spearman rank correlation: *r*_s_ = 0.281, *N* = 521, *P* < 0.001) and temperature ranging from 16.3 to 30.4 °C (Fig. 3); however, the dependency of walking speed from ambient temperature is rather weak. Females did not show any phonotactic behaviour below a temperature of 16 °C.

## Discussion

Numerous studies in the past using different behavioural paradigms provided evidence that the SPL, carrier frequency, or chirp rate influences female choice of crickets in controlled lab conditions (for review see Gerhardt and Huber 2002). A general result was that small differences in these parameters were sufficient to create differences in phonotactic scores to song models. However, female mate choice through phonotaxis has only rarely been observed and quantified in its natural environment. The aim of the present study was, therefore, to describe cricket phonotaxis outdoors and to compare the behavioural data with those obtained under the rather different lab conditions. Although our data confirm the general preference of females in two-choice tests for higher SPL and chirp rate, and for a medium carrier frequency of 4.9 kHz, the field data demonstrated that the necessary differences between two sound stimuli for a significant choice were considerably larger in the natural environment.

### Differences in SPL

In crickets and katydids, differences in the SPL of signals are of relevance both for the outcome of male–male interactions (Dadour and Bailey 1985; Latimer and Schatral 1986) and for preferences exhibited by females in a two-choice situation (Shuvalov and Popov 1973; Gwynne 1982; Doherty 1985; Latimer and Sippel 1987; Bailey and Yeoh 1988; Weber and Thorson 1988; Bailey et al. 1990). One source of evidence for the minimum binaural cues involved for orientation stems from female choice experiments with two sound sources presented from opposite directions (partly summarized by Forrest 1994; Römer et al. 1998; see also Gerhardt and Huber 2002 and literature therein). Depending on species and experimental design, insects prefer one source over the other when the difference is 1–3 dB. The highest sensitivity for differences in SPL was reported when acoustic stimulation was precisely controlled in experiments using dichotic ear stimulation techniques: both grasshoppers and katydids appear able to detect interaural intensity differences (IIDs) as small as 0.5–1 dB that reliably elicit a turn to the louder side (von Helversen and Rheinlaender 1988; Rheinlaender et al. 2005). Similarly, when tested on a fast trackball system, female *G. bimaculatus* exhibit a high sensitivity towards interaural differences in sound intensity and orient towards the louder of two sound sources at IIDs of 1 dB and less (Hedwig and Poulet 2005; Schöneich and Hedwig 2010). Altogether, such experiments reveal the best sensory capacity and resolution for intensity differences of the auditory system available to the insects. However, in two-choice arena trials with female *G. bimaculatus* using an identical stimulus protocol as in the outdoor experiments such small differences in SPL of otherwise identical stimuli have been insufficient for a clear preference for the higher SPL; rather a minimum intensity difference of 3 dB was required for a significant choice of the louder song model (Brunnhofer 2011; SH and HR, unpublished results). However, as the outdoor results show, only an increase in the amplitude difference to 5 dB between the two alternatives resulted in a significant preference for the louder calling song. Despite this significance it is worth mentioning that even in 10 (out of 55) trials females oriented towards, and approached the speaker broadcasting the 5 dB softer song. Thus, for a comparison of results some of the observed differences on the trackball and in dichotic stimulation experiments on the one hand, and in arena experiments on the other, are due to differences between open- and closed-loop conditions, whereas the further increase in the necessary differences outdoors are due to the noisy conditions for sound transmission.

The fact that females require differences in SPL between two song models as large as 5 dB for selective phonotaxis may result from the problems associated with sound propagation parallel to the ground, often referred to as “the forbidden mode of propagation” (Piercy et al. 1977). Indeed, in a neurophysiological study at the same site where our behavioural experiments were performed Kostarakos and Römer (2010) reported strong fluctuations in the intensity gradient of a sound signal over distances between 1 and 16 m. Over the relatively short phonotactic distance of 2 m in the present study such fluctuations appear to be much smaller, although in the summary plot of data from 56 experiments these fluctuations in the intensity gradient will disappear (Fig. 1). It is quite possible though that over larger distances with more pronounced irregularities in the intensity gradient even a 5 dB difference between song alternatives may be insufficient for a significant preference. However, Kostarakos and Römer (2010) also reported strong and irregular variations in directional information on the transmission channel, sometimes providing wrong directional information to the CNS concerning the azimuthal direction of a single sound source, although under identical stimulus configurations in the undistorted laboratory large and correct differences (i.e., the ipsilateral side firing more strongly) have been observed (Kostarakos et al. 2009). Thus, whatever the reason for such discontinuities, a female in an outdoor two-choice experiment may eventually experience either “silent spots” for one or both signals or locations where binaural information regarding the more intense of two stimuli is distorted. This would explain that differences in SPL between two stimuli need to be larger under natural conditions compared to arena or trackball trials. These large differences are even more surprising, since for crickets and katydids a “selective attention” mechanism has been described with the potential to neurally increase the difference in the representation of two stimuli differing by only a few decibels (Pollack 1988; Römer and Krusch 2000). However, given the unpredictable nature of the intensity and directionality fluctuations, the “selective attention” would provide no mechanism to compensate these effects of the transmission channel, since it would operate on SPL differences without discrimination whether the differences represent “true” or “false” information on signal differences.

**Fig. 4:**
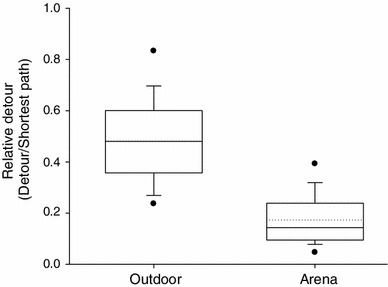
Comparison of detour lengths of phonotactic tracks recorded in outdoor trials and arena trials. In both paradigms two song alternatives only differed in amplitude by 3 dB. Females covered significant longer detours in outdoor experiments (Mann–Whitney rank sum test: *P* ≤ 0.001)

A further reason for differences concerning mate choice decisions based on signal SPL between lab and outdoor experiments is the physical nature of the grassland in which females have to move and orient. Even if females perceive reliable sensory information about the location of the more intense of two stimuli, they might be forced by dense patches of grass or larger obstacles on the ground to move into another, wrong direction. If such misdirected orientation sequences occur repeatedly, a female might deviate from the correct direction up to a point where she is closer to the less intense source, so that distance effects cancel the original SPL differences at the starting point. Even if she eventually arrives at the correct, more intense signal her phonotactic path would be longer. Support for this interpretation provides the calculation of the deviation of the phonotactic approach from the ideal (straight) line towards the speaker. When compared with the same measure obtained from approaches in the arena under similar choice conditions (Brunnhofer 2011), this deviation is significantly reduced under arena conditions (relative detour values of 0.481 for outdoor experiments vs. 0.173 for arena experiments; Mann–Whitney rank sum test: *T* = 964.000, *N* = 24, 29, *P* ≤ 0.001; see Fig. 4).

One of the most obvious reasons for the observed differences in laboratory and field experiments may be background noise and its masking effect for detection of smaller differences in signals (Brumm and Slabbekoorn 2005; Römer 2013). However, the present study area was totally isolated from traffic noise due to surrounding forest and meadows, and experiments were conducted during a time when the only other native cricket species in this area, *G. campestris*, did not sing. Thus, masking noise in the frequency band around 5 kHz was largely absent, except for occasional bird song (see also Kostarakos and Römer 2010).

Finally, one reason for the differences between results obtained outdoors and in the laboratory may be the variation in temperature, which ranged between 16.3 and 30.4 °C in our study compared to arena experiments at 21 °C reported by Brunnhofer (2011). Whereas the increase of walking speed with increasing temperature (Fig. 3) is typical for poikilothermic animals, the effect of temperature or temperature gradients for sound transmission and amplitude fluctuations is unclear (Wiley and Richards 1978). van Staaden and Römer (1997) demonstrated that temperature gradients in the order of 4.5 °C over 10 m may strongly increase the active range of the call of a male bladder grasshopper, and much stronger temperature gradients as a result of sun radiation (about 10 °C over a height of only 30 cm) may occur in the grassland habitat of a field cricket (Römer 2001). Whether such gradients play a role for sound transmission and perception for field crickets needs to be investigated.

### Differences in carrier frequency

When tested with reasonable small increments of frequency differences spanning the natural range of variation in male cricket calling songs, most studies reported stabilizing preferences for the carrier frequency of the call (Ulagaraj and Walker 1975; Thorson et al. 1982; Stout et al. 1983; Shaw and Herlihy 2000; Kostarakos et al. 2008; but see Verburgt and Ferguson 2010 below). Such preferences can usually be explained by the frequency tuning of the peripheral auditory system. In *G. bimaculatus*, the female preference for carrier frequency correlates highly with the tuning of a sensory interneuron (AN1; Kostarakos et al. 2008), known for its importance in cricket phonotaxis (Schildberger and Hörner 1988; Atkins et al. 1992). The tuning of AN1 to a best frequency of 4.9 kHz and the roll-off towards lower and higher frequencies represent a “hard-wired” preference function in *G. bimaculatus*. According to the average tuning of female receivers, frequency differences between two calling songs can be translated into differences in the perceived sound pressure level (Kostarakos et al. 2009). As shown in their study, a frequency difference of 200 Hz (4.9 vs. 4.7 kHz) equals an intensity difference of 2.6 dB, and a difference of 400 Hz (4.9 vs. 5.3 kHz) is equivalent to an intensity difference of 3.75 dB. In both of these choice trials, our outdoor results revealed no significant preference for the signal at the best frequency of hearing (4.9 kHz), which is consistent with the results obtained with signal differences in SPL, where a minimum difference of 5 dB was necessary for selective female phonotaxis. Again, the significant preferences observed in a choice for the standard model at 4.9 kHz versus alternatives differing by 500 Hz and 900 Hz (4.4 or 4.0 kHz, respectively) result in perceived level differences of 9.6 and 23.7 dB, clearly well above the level difference of 5 dB.

Preferences of females for the carrier frequency of the male call are often examined in the context of preferences for indicators of body size, due to the negative correlation of male size and carrier frequency as a result of sound production, where larger resonators produce lower frequencies (for review see Brown 1999; Gerhardt and Huber 2002). However, whereas some studies confirmed female preferences for lower than average carrier frequency in crickets (Simmons and Ritchie 1996; Brown et al. 1996; Scheuber et al. 2004), others do either find no preference for carrier frequency in the same species (Deb et al. 2012), or even a preference for higher over lower frequencies (Kostarakos et al. 2008; the present study). The shape and steepness of the peripheral tuning also determine the sensitivity to small variations in male carrier frequency: behavioural tuning curves in the field crickets *Teleogryllus commodus* and *T. oceanicus* fall off more sharply at low frequencies compared to high frequencies (Hennig and Weber 1997). Notably, in their study on *Oecanthus henryi*, Deb et al. (2012) carefully examined these preferences at two SPL values close to hearing threshold and well above threshold, as well as with increments of frequency differences spanning the large range of frequencies to which the system is tuned (2.5–4.5 kHz; Mhatre et al. 2011). Despite the lack of female preference for this call character which is negatively correlated with body size, females nevertheless demonstrated a preference for larger males in several parameters of their mating behaviour. Thus, the finding that larger males have a mating advantage in the field (Simmons 1986) may not be attributed to choice based on acoustic signal parameters, but on close range cues (Thomas and Simmons 2009; Simmons et al. 2013).

Whereas our results on selective phonotaxis in the field are consistent with earlier studies obtained in the lab demonstrating stabilizing selection for a carrier frequency which corresponds to the best frequency of hearing, Verburgt and Ferguson (2010) argued for the same species that females are unable in detecting differences in song carrier frequency of males. However, their results are confounded by the fact that one cannot judge the discriminatory potential of receivers from preference functions obtained in no-choice trials, since females will track a male calling song for a large range of carrier frequency, given the SPL is well above the behavioural threshold for phonotaxis. Indeed, no-choice trials for carrier frequencies of 4.4, 4.9 and 5.2 kHz did not reveal any significant difference in the absolute amount of lateral steering on a trackball system (Hirtenlehner et al. 2013). In a choice situation, however, the relative intensities of the two signals become highly relevant as a result of the underlying tuning of neuronal elements (Kostarakos et al. 2008). Brown et al. (1996) also found a preference for carrier frequency in female *O. nigricornis* only when they had the opportunity to compare calls broadcast simultaneously.

### Differences in chirp rate

Numerous species of insects and anurans demonstrate female preferences for higher call or chirp rates over lower ones, a parameter which is most variable among individual and populations (Ryan and Keddy-Hector 1992; review in Gerhardt and Huber 2002). Assessing the sensory information for such a dynamic song character poses the problem that the actual value of a difference between two or more signals accumulates only slowly over time. The smaller the difference in chirp rate, the longer it should take for evaluating this information. This creates a dilemma known as the speed–accuracy trade-off (Wickelgren 1977; Chittka et al. 2009). Indeed, under open-loop conditions on a trackball system female *G. bimaculatus* significantly preferred the higher chirp rate when the minimum difference was only 20 chirps/min, and the lateral steering into the direction of the higher rate reached a predefined threshold earlier with higher rate differences (Trobe et al. 2011). We are unaware of any field studies addressing mate choice for this dynamic property of the calling song alone, but under field conditions, this difference was not sufficient for a preference, and female crickets required a chirp rate difference of 50 chirps/min (150 vs. 100) for a reliable decision for higher rates. During the average time needed by a female over the phonotactic path towards the preferred speaker (84.6 s) she experienced about 70 chirps more through the signal with the higher chirp rate. Although such large differences between male calling songs may occur, they nevertheless present extremes in the distribution, and may thus not occur very frequently.

## Conclusion

Our data on acoustic preferences of female crickets examined under conditions of natural grassland have shown that the small differences in signal parameters which are sufficient in two-choice trials under various laboratory settings do not provide reliable information outdoors. Similar to the discussion about the importance of fluctuating asymmetry, where many of the reported preferences for symmetrical traits have been obtained with trait values very rarely experienced in nature (Swaddle 1999), the present finding has important consequences when considering the strength of sexual selection through female choice. However, we are aware of the problem that a direct comparison between results of the numerous studies on acoustic insects should be treated with caution. They differ in many aspects, such as in their open or closed-loop condition, whether female preferences are tested in no-choice or choice trials, whether the choice was simultaneous or sequential, or whether the choice was based on single or multiple sound sources. A further difference relates to the use of multisensory, in particular visual, information, since our outdoor experiments were performed during the day, and females could potentially use visual landmarks in combination with acoustic directional information, whereas lab experiments are usually conducted in the dark. Moreover, in arena trials the distance covered by females from the starting point to the target was often less than 50 cm (Mhatre and Balakrishnan 2007, 2008), giving the female less freedom to redirect her path because intensity effects through distance become most important for the decision. Studies also vary considerably in their spatial arrangement of the sound sources, placed either from opposite ends of the arena (separation 180°) or values between 50 and 90°, with consequences for the amount of directional information for the alternative stimuli.

## Abbreviations

CF: Carrier frequency
GLMM: Generalized linear mixed model
IID: Interaural intensity difference
JMD: Just meaningful difference
JND: Just noticeable difference
SPL: Sound pressure level
